# A Rectal Mass That Deceived: Solitary Plasmacytoma Masquerading as Mantle Cell Lymphoma With Subsequent Progression to Multiple Myeloma

**DOI:** 10.7759/cureus.88075

**Published:** 2025-07-16

**Authors:** Milaris M Sanchez-Cordero, Josean Rosado Rivera, Leanette Guzman, Santa Merle

**Affiliations:** 1 Internal Medicine, Mayagüez Medical Center, Mayagüez, PRI; 2 Hematology and Oncology, Mayagüez Medical Center, Mayagüez, PRI

**Keywords:** cyclin d1, immunophenotyping, mantle cell lymphoma, multiple myeloma, rectal neoplasms, solitary plasmacytoma

## Abstract

Solitary extramedullary plasmacytomas (SEPs) are rare plasma cell tumors, particularly when located in the gastrointestinal tract. Misdiagnosis may occur due to overlapping histological and immunophenotypic features with other hematologic malignancies, such as mantle cell lymphoma (MCL). We present the case of a 63-year-old female patient initially diagnosed with MCL based on rectal mass biopsy, who underwent lymphoma-directed therapy. Surgical excision and further histopathological evaluation revised the diagnosis to SEP. Over time, the patient developed serum monoclonal gammopathy and progressed to multiple myeloma (MM). This case highlights the diagnostic pitfalls of rectal lymphoid lesions, the value of surgical pathology, and the potential for SEPs to evolve into systemic plasma cell neoplasia.

## Introduction

Solitary extramedullary plasmacytoma (SEP) is a rare variant of plasma cell dyscrasia that presents as a localized clonal plasma cell proliferation outside of the bone marrow. SEPs account for less than 5% of all plasma cell neoplasms, and gastrointestinal (GI) involvement is even more uncommon, representing fewer than 10% of all cases [1,2]. When SEPs occur in the GI tract, the stomach, small intestine, and colon are more frequently affected than the rectum [3]. Rectal plasmacytomas are exceedingly rare, with few documented cases in the literature [4].

Differentiating SEP from lymphomas such as mantle cell lymphoma (MCL) is diagnostically challenging due to overlapping histological and immunophenotypic features. MCL is a B-cell non-Hodgkin lymphoma characterized by Cyclin D1 overexpression as a result of the t(11;14)(q13;q32) translocation [5]. Cyclin D1 expression is a hallmark of MCL; however, it can occasionally be observed in plasma cell neoplasms, including plasma cell myeloma and SEP, contributing to diagnostic confusion [6].

The initial misdiagnosis of SEP as MCL may lead to inappropriate therapy and a delay in initiating plasma cell-targeted treatment. This case underscores the importance of comprehensive immunohistochemical panels and, when needed, full-thickness surgical specimens for accurate diagnosis. Additionally, it highlights the clinical relevance of disease progression from SEP to systemic involvement as multiple myeloma (MM), a transformation observed in 50-70% of SEP cases over time [7,8].

## Case presentation

A 63-year-old female patient presented with rectal pain, tenesmus, and altered bowel habits. Colonoscopy identified a rectal mass, and initial biopsy showed atypical lymphoid infiltrates. Immunohistochemistry revealed positivity for Cyclin D1, CD5, CD20, and a high Ki-67 index, leading to a preliminary diagnosis of MCL. The patient was started on chemotherapy accordingly.

**Figure 1:**
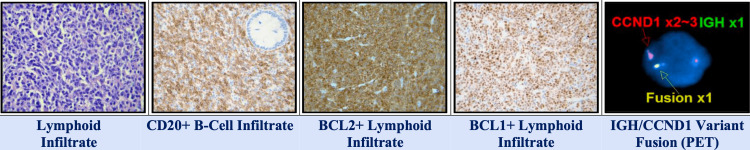
Colon and rectosigmoid segments Histologic sections of colonic mucosa show diffuse infiltration by small to intermediate-sized neoplastic lymphoid cells. Immunohistochemical staining demonstrates that the infiltrate is positive for CD20, BCL1 (Cyclin D1), and BCL2

Due to an incomplete clinical response and persistent symptoms, surgical excision of the mass was performed. Final histopathology of the excised specimen demonstrated a dense monoclonal plasma cell infiltrate. Immunostaining was positive for CD138 and CD56 and showed Kappa light chain restriction. Markers including CD20 (+), CD5 (-), and Cyclin D1 (-) (Figure 1). These findings were diagnostic of SEP (Table 1).

**Table 1:** Immunohistochemistry results of colon and rectosigmoid segments BCL: B-cell lymphoma; DLBCL: diffuse large B-cell lymphoma

Antibody	Specificity	Result
BCL6	Follicular center	Negative
BLC2	Mature T-lymphocytes and Various B-cell lymphomas	Positive
CD20	Pan B-lymphocytes	Positive
CD10	B-lymphoblasts, follicular center cells & grans	Negative
BCL1	Mantle cell lymphoma	Positive
CD5	Pan T-lymphocytes	Negative
CD23	Follicular dendritic cells (FDC)	Negative
CD21	Follicular dendritic cells	Negative
CD30 (Ki-1)	Immunoblasts and Hodgkin Reed-Sternberg cells	Negative
CD3	Mature T-lymphocytes	Negative
Ki-67	Proliferative Index	Low (10-20%)
c-MYC	DLBCL subsets	Negative

**Figure 2:**
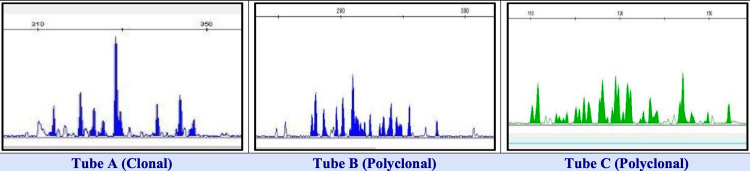
Molecular diagnosis (IGH) IGH: immunoglobulin heavy chain Colon-rectosigmoid segments: (+) for clonal B-cell IGH gene rearrangement.

Subsequent workup included serum protein electrophoresis (SPEP), which showed an M-spike, and a serum free light chain assay that demonstrated a significantly abnormal Kappa/Lambda ratio. Initial bone marrow biopsy was negative for plasma cell infiltration (Table 2, Figure 2).

**Table 2:** FISH diagnosis FISH: fluorescence in situ hybridization FISH is performed on paraffin-embedded tissue section. The positive IGH/CCND1 translocation by supports the diagnosis of mantle cell lymphoma

Locus	Probe	Result	Cell counted
11q13.3/14q32.33	CCND1/IGH	Abnormal/IGH/CCND1 variant translocation (88%)	100

**Figure 3:**
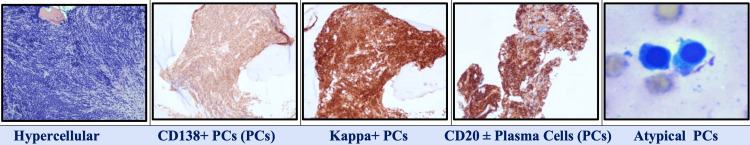
Bone marrow biopsy and aspirate findings BCL: B-cell lymphoma Bone marrow biopsy reveals a markedly hypercellular marrow with diffuse infiltration by plasma cells, comprising approximately 90% of the cellular population. Immunohistochemical staining shows strong CD138 positivity, partial CD20 expression, and BCL1 (Cyclin D1) positivity, consistent with a clonal IgG/kappa-restricted plasma cell neoplasm The aspirate is aspicular and moderately hemodiluted, demonstrating 60% markedly atypical plasma cells.

However, six months later, a repeat bone marrow biopsy revealed clonal plasma cells to be ≥10%. Positron emission tomography/computed tomography (PET/CT) imaging did not identify other lesions. The patient was diagnosed with evolving MM and treated with bortezomib, lenalidomide, and dexamethasone (Figure 3, Table 3).

**Table 3:** Bone marrow: markedly atypical plasma cells This result is based on 200 cells counted. The aspirate smears are aspicular, moderately hemodiluted with marked preparational artifacts. They show 60% atypical plasma cells. The sparse hematopoietic precursors show orderly maturation and no dyspoiesis or increased blasts

Cell type	Result (%)	Reference range (%)
Pronormoblast	0.0	0-1.0
Basophilic normoblast	0.0	0-5.0
Polychromatic normoblast	0.0	6.0-16.0
Orthochromatic normoblast	2.5	4.0-18.0
Lymphocyte	6.5	3.0-20.0
Plasma cell	60.0	1.0-4.0
Promonocyte	0.0	0-2.0
Monocyte	0.0	1.0-4.0
Myeloblast	1.0	0-1.0
Promyelocyte	0.0	2.0-4.0
Myelocyte		
Granulocyte	2.5	5.0-19.0
Eosinophil	0.0	0.5-3.0
Basophil	0.0	0-1.0
Metamyelocyte	4.5	12.0-22.0
Band	10.5	8.0-16.0
Segmented		
Granulocyte	12.5	7.0-22.0
Eosinophil	0.0	0.5-4.0
Basophil	0.0	0-1.0
Megakaryocytes: not adequately represented		
Iron stain: cannot be assessed (aspicular with no ring sideroblasts)		

**Figure 4:**
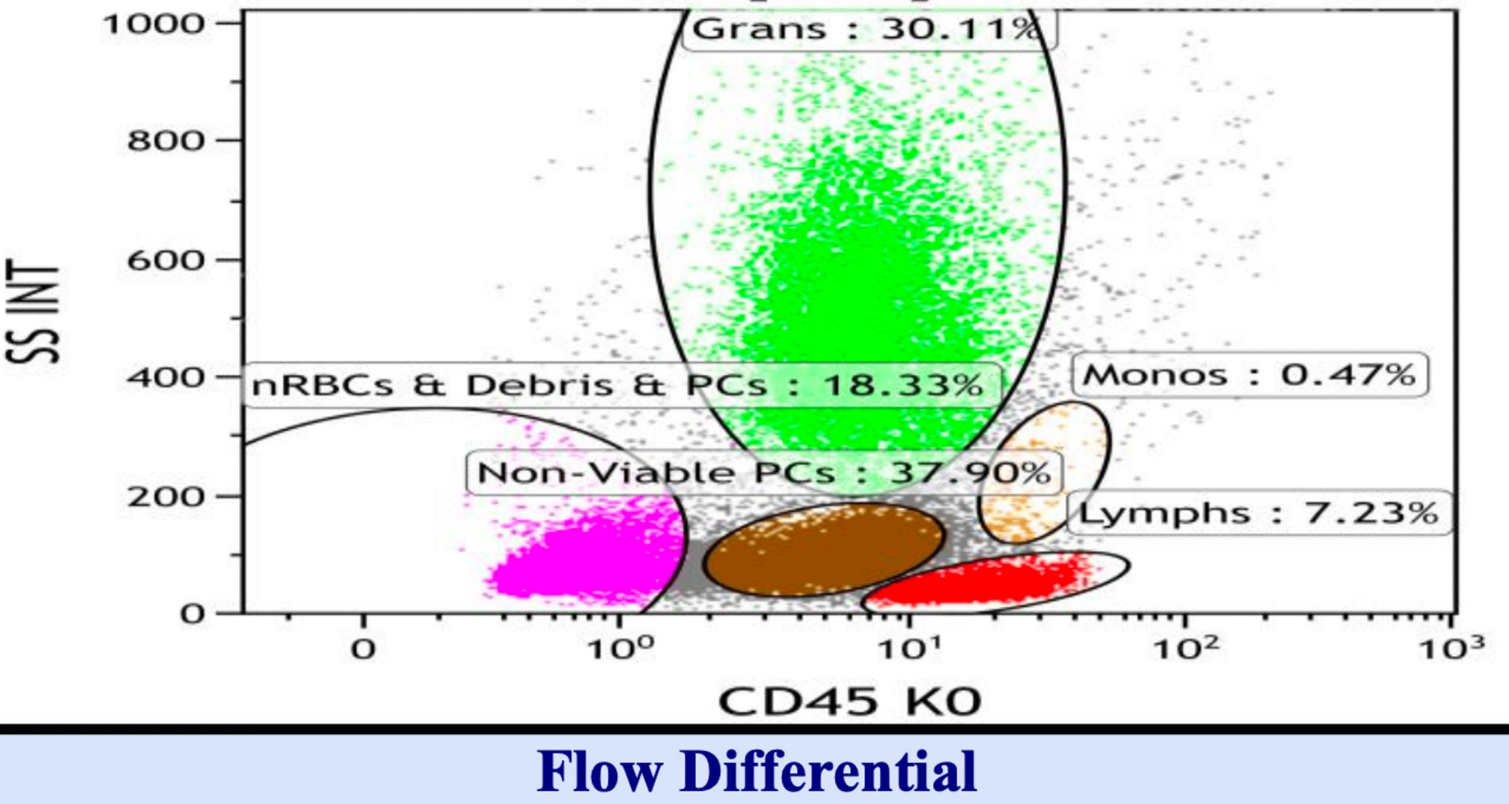
Flow cytometry 1A

**Figure 5:**
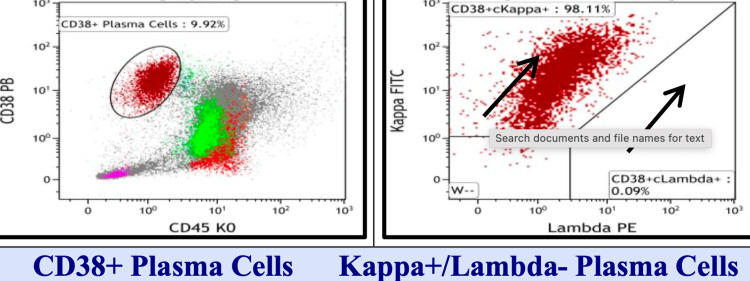
Flow cytometry 1B

**Figure 6:**
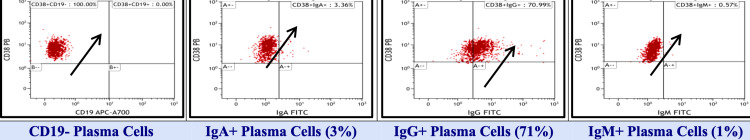
Flow cytometry 1C

Additional evaluations from April to November 2023 showed progressive increases in serum free Kappa chains (from 248.19 to 2107.20 mg/L), a Kappa/Lambda ratio rising to 114.9, and positive Bence Jones proteinuria. By September 6, 2024, the patient developed a retromandibular mass. Biopsy confirmed extramedullary plasmacytoma with CD138+, Cyclin D1+, MUM1+, and Kappa-restricted cells (Tables 4-5, Figures 4-6).

**Table 4:** Flow cytometry diagnosis: bone marrow aspirate NK cell: natural killer cell IgG/Kappa monoclonal plasma cells with aberrant phenotype: the smear from the tube is moderately hemodiluted with sparse hematopoietic precursors. By flow, the CD38+ plasma cells (10%), although increased, are underrepresented due to hemodilution and lysis during processing. They have an IgG/kappa monoclonal phenotype with aberrant loss of CD19 and CD27. The lymphocytes (7%) include 48% mature T-cells with a mildly reactive inverted CD4/CD8 ratio of 3:4 (normal 0.8-3:1), 54% polyclonal B-cells, and 2% natural killer cells. A 38% population of nonviable plasma cells is detected. The total events analyzed are 40,279

Analysis		Gatting strategy	CD45 and side scatter
Specimen	Bone marrow	Viability	51% (Normal >80%)
Gated population	Lymphs	Plasma cells	Large B-lymphs
Gated cells	7%	10%	1%
B-cell markers			
CD19	54%		
CD20	53%		
CD10	5%		
CD23	6%		
CD19/CD5	<1%		
CD19/CD38	8%		
CD38	4%		
CD79b	40%		
CD103	1		
CD19/CD123	<1%		
BCL2	<1%		
CD19/Kappa	67%		65%
CD19/Lambda	33%		25%
CD43	<1%		
CD200	41%		
T-cell markers			
CD3	48%		
CD4	20%		
CD8	26%		
CD2	54%		
CD5	47%		
CD7	57%		
NK cell markers			
CD56	9%		
CD16	2%		
CD57	8%		
Plasma cell markers			
CD38/CD138		99%	
CD38/CD56		<1%	
CD38/CD19		<1%	
CD38/CD27		<1%	
CD38/CD117		<1%	
CD38/CD20		3%	
CD38/Kappa		98%	
CD38/Lambda		<1%	
CD38/IgA		<1%	
CD38/IgG		5%	
CD38/IgM		<1%	

**Table 5:** FISH diagnosis: bone marrow aspirate FISH: fluorescence in situ hybridization Standard risk myeloma with IGH/CCND1 translocation and monosomy 13

Locus	Probe	Result	Result	Cell counted
1p36.31/1q25.3	1p36/1q25.	ZytoVision #Z-2075-200	Normal	200
9q12	CEN9	ZytoVision #Z-2067-200	Normal	200
11q13.3/14q32.33	CCND1/IGH	ZytoVision #Z-2125-200	Abnormal/IGH/CCND1 variant translocation (82%)	200
13q142/13q34	D13S319/13q34 (G)	ZytoVision #Z-2280--50	Abnormal/monosomy 13 (87%)	200
17p13.1/13p11.1-q11.1	TP53/CEN17	ZytoVision #Z-2153-200	Normal	200

**Figure 7:**
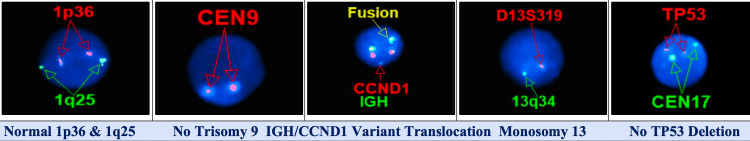
Fluorescence in situ hybridization (FISH) results: bone marrow aspirate FISH analysis performed on conventional bone marrow preparation reveals a positive IGH/CCND1 (t[11;14]) translocation and monosomy 13. In the context of multiple myeloma, the presence of IGH/CCND1 is associated with standard-risk disease based on the International Cytogenetic Risk Stratification System. Monosomy 13 is of uncertain prognostic significance

These were negative for CD19, CD20, CD45, and Lambda, supporting clonal progression from MCL to plasma cell neoplasm (Tables 6-8).

**Table 6:** Timeline of disease progression and key pathology FISH: fluorescence in situ hybridization This table outlines the chronological sequence of clinical events, diagnoses, and corresponding pathology results, including transitions from solitary plasmacytoma to overt multiple myeloma

Date	Finding	Interpretation
May 29, 2020	Biopsy: Cyclin D1+, CD20+, BCL1+ MCL in sigmoid colon, IGH/CCND1 by FISH	Diagnosis: mantle cell lymphoma (MCL)
2021-2022	Serial colon biopsies with persistent infiltration, evolving morphology	Reclassified as CD20+ plasmacytoid neoplasm
Oct 13, 2022	Bone marrow: 90% cellularity, CD138+, BCL1+, Kappa restriction	Plasma cell neoplasm confirmed via flow cytometry (CD19-/CD27-)
Apr-Nov 2023	Free Kappa ↑ from 248.19 → 2107.20 mg/L; Kappa/Lambda ratio ↑ to 114.9; Bence Jones proteinuria	Confirms light-chain multiple myeloma secretory phenotype
Sep 6, 2024	Retromandibular mass: CD138+, Cyclin D1+, Kappa+, Lambda-, CD19/20/45-	Extramedullary plasmacytoma with clonal evolution

**Table 7:** Summary of serum and urine findings SPEP: serum protein electrophoresis; AL: amyloid light chain Key laboratory data, including SPEP), immunofixation, and free light chain ratios that supported progression to plasma cell dyscrasia

Date	Test	Result	Interpretation
Apr 20, 2021	Immunofixation (urine)	Two monoclonal Kappa light chains	Suggestive of plasma cell disorder (e.g., light-chain MM or AL amyloidosis)
Apr 23, 2023	Free light chains (serum)	Kappa 248.19 mg/L, K/L ratio = 25.1	Strongly suggestive of monoclonal proliferation
Nov 2023	Free light chains (serum)	Kappa 2107.20 mg/L, K/L ratio = 114.9	Aggressive clonal plasma cell proliferation
Nov 2023	Immunofixation (urine)	Positive; proteinuria 59.5 mg/dL	Bence Jones confirmed
Nov 29, 2023	SPEP	M-spike 2.67%, Gamma 0.15 g/dL	Early persistent monoclonal gammopathy

**Table 8:** Key diagnostic criteria and transitions Comparison of initial diagnostic features with evolving clinical and histopathologic criteria, highlighting the transition from presumed mantle cell lymphoma to confirmed solitary plasmacytoma and eventually multiple myeloma

Phase	Key markers	Interpretation
Initial (2020)	Cyclin D1+, CD20+, t(11;14)	Mantle cell lymphoma
Transition (2021-2022)	Persistent CD20+, evolving morphology	Suspicion of plasmacytic differentiation
Progression (2022)	CD138+, Kappa+, CD19-	Monoclonal plasma cell neoplasm
Light-chain MM (2023-2024)	↑ Free Kappa, ↑ K/L ratio, Bence Jones, ↓ gamma fraction	Confirmed light-chain secretory multiple myeloma
Extramedullary involvement	CD138+, Cyclin D1+, Kappa+, Lambda-, CD19/20/45- (retromass)	Clonal progression to extramedullary plasmacytoma

Despite multiple lines of therapy including bortezomib, daratumumab, cyclophosphamide, Kyprolis, and pomalidomide, the disease progressed biochemically and extramedullary, with persistent Kappa chain secretion and need for morphine-based analgesia.

## Discussion

This case illustrates the diagnostic complexity associated with rectal lymphoid or plasma cell neoplasms. GI plasmacytomas are rare and often misclassified due to their overlapping morphology and immunophenotype with lymphomas, especially MCL [3,4,6]. Cyclin D1 positivity, although characteristic of MCL, may be expressed in plasma cell neoplasms, contributing to diagnostic error [5,6].

Accurate distinction between SEP and MCL is essential, given the vastly different therapeutic approaches. In our case, the patient initially received lymphoma-directed therapy before surgical resection, and expanded immunophenotyping clarified the true diagnosis. Misclassification can lead to suboptimal treatment and delays in appropriate systemic therapy for plasma cell neoplasms.

According to the International Myeloma Working Group (IMWG), diagnosis of MM requires ≥10% clonal plasma cells in bone marrow or biopsy-proven plasmacytoma with CRAB features (hypercalcemia, renal dysfunction, anemia, bone lesions) or specific biomarkers such as high serum free light chain ratios (>100) [9,10]. Our patient met multiple criteria: increasing free Kappa chains, high Kappa/Lambda ratio, Bence Jones proteinuria, and ultimately, clonal bone marrow plasma cells and extramedullary progression. These changes document a clear trajectory from SEP to MM.

Studies have shown that SEP has a five-year progression risk to MM ranging from 30% to 70% depending on site, size, and baseline laboratory values [7,8]. GI tract SEPs, although rare, should be closely monitored with serum free light chains, SPEP, and periodic bone marrow evaluation.

In addition to Cyclin D1, the use of markers such as CD138, CD38, and light chain restriction by immunohistochemistry and flow cytometry is critical in characterizing plasma cell disorders [6]. In ambiguous cases, fluorescence in situ hybridization (FISH) and molecular studies can further delineate lymphoid from plasma cell malignancies.

## Conclusions

This case is notable for the clinical rarity of a solitary plasmacytoma located in the rectum, an uncommon site for extramedullary plasma cell neoplasms. The definitive diagnosis was initially delayed because the patient declined a bone marrow biopsy, leading to the continuation of lymphoma-directed therapy under the presumption of MCL. However, due to a lack of response and persistent disease, surgical resection and full immunophenotypic workup were pursued, ultimately revealing solitary plasmacytoma. This case underscores the importance of comprehensive diagnostic evaluation in Cyclin D1-positive rectal masses, particularly when there is an atypical response to standard lymphoma regimens. In such scenarios, clinicians must maintain a high index of suspicion for plasma cell dyscrasias, even in unusual anatomical sites.

Accurate immunophenotyping, molecular testing (including FISH), and surgical pathology are essential tools that can prevent misdiagnosis and facilitate timely, targeted treatment. Furthermore, this case highlights the potential for SEPs to evolve into systemic MM, justifying the need for long-term surveillance with serial imaging, free light chain monitoring, and bone marrow reassessment. This case contributes to the limited but growing literature on rectal plasmacytomas and reinforces the importance of integrating pathology, molecular genetics, and clinical acumen in the management of rare plasma cell neoplasms.
